# Construction of a Nano-Controlled Release Methotrexate Delivery System for the Treatment of Rheumatoid Arthritis by Local Percutaneous Administration

**DOI:** 10.3390/nano11112812

**Published:** 2021-10-23

**Authors:** Tingting Guo, Xu Kang, Sifan Ren, Xianjin Ouyang, Mingming Chang

**Affiliations:** 1 College of Bioscience and Resource Environment, Beijing University of Agriculture, Beijing 102206, China; g.t0103@icloud.com (T.G.); kangkangkangxu@gmail.com (X.K.); sifanr@outlook.com (S.R.); txiongla0@gmail.com (X.O.); 2 Key Laboratory of Urban Agriculture (North China) Ministry of Agriculture, Beijing University of Agriculture, Beijing 102206, China

**Keywords:** methotrexate, local percutaneous administration, polydopamine, pH-sensitive, rheumatoid arthritis

## Abstract

A drug delivery system was specifically designed for the treatment of rheumatoid arthritis (RA) by local percutaneous administration and the nano-controlled release of methotrexate (MTX). The release behavior of MTX from the synthesized MTX-mSiO_2_@PDA system was investigated in vitro and in vivo. The obtained results show that after 48 h, twice as much MTX (cumulative amount) is released at pH 5.5 than at pH 7.4. This suggests that the MTX-mSiO_2_@PDA system exhibits a good pH sensitivity. In vitro local percutaneous administration experiments revealed that the cumulative amount of MTX transferred from MTX-mSiO_2_@PDA to pH 5.0 receptor fluid through the whole skin was approximately three times greater than the amount transferred to pH 7.4 receptor fluid after 24 h. Moreover, in vivo experiments conducted on a complete induced arthritis (CIA) model in DBA/1 mice demonstrated that the thickness of a mouse’s toes decreases to nearly 65% of the initial level after 27 days of local percutaneous MTX-mSiO_2_@PDA administration. Compared to the mice directly injected with MTX, those administered with MTX-mSiO_2_@PDA by local percutaneous application exhibit much lower toe thickness deviation, which indicates that the latter group experiences a better cure stability. Overall, these results demonstrate that the local percutaneous administration of MTX delivery systems characterized by nano-controlled release may play an important role in RA therapy.

## 1. Introduction

Rheumatoid arthritis (RA) is a chronic autoimmune disease that is mainly caused by joint synovial inflammation [1,2] and that induces cartilage and bone damage. Thus far, there is no cure for RA [3]; however, methotrexate (MTX) is an effective and highly tolerable drug that is commonly used to treat this disease [4]. This drug regulates the abnormal immunity of patients, significantly reduces cartilage destruction and bone erosion, controls increases in bone lesions, prevents or delays joint damage, and inhibits disability [5,6]. Despite the high efficiency of MTX, its clinical application is limited by its systemic complications and side effects associated with the long-term administration of high doses of the drug, including femoral head necrosis, hypertension, and weight gain. In an attempt to reduce these side effects, patients have been given repeated intra-articular MTX injections in their damaged joints, but many of them did not adapt well to such treatment [7,8]. Therefore, it is of great significance to develop safe and effective MTX delivery systems for the treatment of RA.

Local percutaneous administration involves the application of drugs on the skin and their absorption, at a certain rate, into the local tissue [9,10,11]. In the last few years, many drugs and delivery systems have been constructed for the treatment of RA via local percutaneous administration [12,13,14,15]. I.A. Tekko’s group [16] developed microneedle arrays for the painless delivery of MTX via the skin’s stratum corneum barrier. This delivery system supplies large quantities of the drug and is suitable for use in juvenile idiopathic arthritis patients. Neeraj K. Garg’s group [17] prepared lipid nanoparticles that can efficiently deliver MTX, with minimal adverse effects, via percutaneous application. These nanoparticles can help overcome the limitation of poor MTX solubility and the requirement of long-term application of high drug doses [18,19]. In addition, they can enhance the percutaneous absorption of insoluble drugs, thereby increasing the drug delivery rate [20,21].

Mesoporous materials are widely used in drug delivery systems due to their high biocompatibility and controllable morphology [22,23]. In particular, mesoporous silica nanoparticles (mSiO_2_) are considered an ideal choice for the construction of nano-release systems, as they can increase the content of a drug in solution by increasing its solubility [24,25,26]. The nanoscale pores in mesoporous materials enhance the release rate of insoluble drugs by preventing the formation of drug molecule crystals and maintaining an amorphous state [27]. In 2014, Zhao et al. [28] successfully prepared a novel biocompatible mSiO_2_ material with an adjustable pore size using cetyltrimethylammonium chloride (CTAC) as a template. Based on the results reported by the authors, this material constitutes an ideal system for nano-controlled drug release. As drug delivery carriers, mesoporous silica nanoparticles are widely studied for use in cancer therapy. Using mesoporous silica nanoparticles as carriers for rheumatoid arthritis local percutaneous administration might improve the MTX cure effect.

Dopamine (DA) is a neurotransmitter that plays very important roles in the brain and body and constitutes about 80% of the catecholamine content in the brain. This is a highly biocompatible compound. Under alkaline conditions, the catechol groups in DA are converted to chinone, leading to the formation of polymeric PDA thin films that can be used to cover the surfaces of various materials, including drugs [29]. At pH < 7.0, PDA undergoes gradual degradation, thereby releasing enclosed drug molecules. To control drug release, Qi Chen’s group [30] previously developed organic/inorganic hybridized hollow mSiO_2_ with a PDA-modified surface. Similarly, Yi Wei et al. [31] covered the DOX drug delivery system with PDA to enhance DOX targeting. Although PDA is widely applied in injectable delivery systems, the application of PDA-modified mSiO_2_ in percutaneous delivery systems has not yet been reported. In this study, we design a pH-sensitive, nano-controlled release system for the delivery of MTX (MTX-mSiO_2_@PDA) via percutaneous application. This system is prepared by encapsulating MTX in the pores of the mesoporous material, which is covered by a “gated” channel. After percutaneous application, the prepared material passes through the skin into the inflamed tissue where the pH is less than 7.0. The acidic conditions trigger the degradation of the PDA “gate switch”, resulting in the release of MTX. Considering that the stimulus-response, controlled-release drug delivery system proposed herein achieves local drug administration, it is expected that this system will reduce the toxicity and increase the effectiveness of MTX as a rheumatoid arthritis therapeutic treatment.

## 2. Materials and Methods

### 2.1. Cell Lines and Animals

RAW 264.7 cells (leukemia cells in mouse macrophage) in fresh DMEM supplemented with 10% FBS (fetal bovine serum) were purchased from Cell Resource Center, IBMS, CAMS/PUMC. The medium was refreshed every day during the cell culture protocol.

SD rats and DBA/1 mice were provided by the Academy of Military Science of the People’s Liberation Army in China. The mice were acclimated to the environment in standard rat cages, where they were supplied with pure water and rat food for three days before the experiment. All experimental procedures were performed according to the International Guidelines for the Care and Use of Laboratory Animals and approved by the Experiment Animal Administrative Committee of the Beijing University of Agriculture.

### 2.2. Materials

MTX was purchased from Xingheng Chemical Technology Co., Ltd. (Wuhan, China), whereas dopamine (DA) was acquired from Biotopped (Beijing, China). Cetyltrimethylammonium chloride (CTAC), trifluoroacetic acid (TFA), and tetraethyl orthosilicate (TEOS) were bought from Sigma-Aldrich (St. Louis, MO, USA), MREDA (Beijing, China), and Bailingwei Technology Co., Ltd. (Beijing, China), respectively. Triethanolamine (TEA) and cyclohexane were purchased from Xilong Chemical Co., Ltd. (Foshan, China). Chromatography-grade acetonitrile, DMEM, and FBS were purchased from Thermo Fisher (Waltham, MA, USA). All other reagents were analytically pure.

### 2.3. Synthesis of MTX-mSiO_2_@PDA

#### 2.3.1. Loading of MTX into mSiO_2_

MTX (5 mg) was fully dissolved in 50 μL of dimethyl sulfoxide (DMSO) and then dropped in a solution of 1 mL of PBS containing 5 mg of mSiO_2_ (pH 7.4). The mixture was shaken for 24 h at room temperature in the dark and then centrifugated to collect the MTX-mSiO_2_ nanoparticles. The centrifugation process was repeated many times to ensure that any residual MTX had been washed off. Finally, the load and packet rates were calculated.

#### 2.3.2. Construction of MTX-mSiO_2_@PDA

DA (10 mg) and MTX-mSiO_2_ (10 mg) were dispersed in PBS at pH 8.5. The mixture was stirred in the dark for 24 h, then it was centrifuged to flush the unpolymerized DA with PBS (10 mL) and collect the MTX-mSiO_2_@PDA material.

### 2.4. Confocal Laser Scanning Microscopy Analysis of Cellular Uptake

RAW 264.7 cells were seeded and cultured overnight in a 6-well plate at a density of 105 cells/well. The cultured cells were subsequently incubated with 5 μg of FITC equivalent/mL solutions of FITC-mSiO_2_@PDA or FITC-mSiO_2_ in fresh DMEM (10% FBS) for 0.5, 1.0, or 12.0 h. Afterward, the unbound samples were washed off with PBS and fixed with 4% (*w*/*v*) cold paraformaldehyde for 10 min. The cells were then washed three times with PBS and viewed using an LSM710 confocal laser scanning microscope (Carl Zeiss, Jena, Germany). The excitation and emission wavelengths of FITC were 488 and 519 nm, respectively. The recorded microscopic images were analyzed by the Zen_2012 (blue edition) software.

### 2.5. In Vitro Antiproliferative Activity

RAW 264.7 cells were seeded in a 96-well plate at a density of 5 × 10^3^ cells/well. The cells were divided into four groups, with 0.01, 0.1, or 1 mmol/L MTX added to the first group and MTX-mSiO_2_@PDA containing 0.01, 0.1, or 1 mmol/L MTX added to the second group. As for the third and fourth groups of cells, they were administered with mSiO_2_ and mSiO_2_@PDA, respectively, at the concentrations of 4.54, 45.4, or 454 mg/L. The samples were incubated in fresh DMEM (10% FBS) for 24 h, and the activity of cells was examined by an MTT assay. Three parallel experiments were conducted at each concentration.

### 2.6. Release Behaviors of MTX-mSiO_2_ and MTX-mSiO_2_@PDA

To assess the release behaviors of MTX-mSiO_2_ and MTX-mSiO_2_@PDA, the nanoparticles were dispersed in 1 mL of PBS at different pH levels (5.0, 6.5, 7.4), and the dispersions were shaken at room temperature in the dark. At 0, 0.5, 2, 4, 8, 24, and 48 h, 200 µL samples of the release solution were collected and replaced with 200 µL of PBS at the same pH. The concentration of MTX in the collected samples was determined. At the end of the experiment, the MTX-mSiO_2_ nanoparticles were removed from the release solution and dispersed in 200 µL of DMSO. The mixture was centrifuged to release any remaining MTX in mSiO_2_, and the process was repeated three times. As for the MTX-mSiO_2_@PDA nanoparticles, they were also collected at the end of the experiment; however, unlike the MTX-mSiO_2_ nanoparticles, they were added to 50 µL of hydrochloric acid to sufficiently degrade the PDA film. The MTX released upon PDA degradation was subsequently dissolved in 200 µL of DMSO and treated as the drug released from MTX-mSiO_2_. Finally, the cumulative release rates of MTX from MTX-mSiO_2_ and MTX-mSiO_2_@PDA were calculated.

### 2.7. In Vitro Percutaneous Experiment

After sacrificing the SD rats with ethyl ether, their abdominal skin was cut off. The hairs on the cut skin samples were gently removed using an electric razor, or they were peeled with tape 15 to 20 times to obtain a stripped stratum corneum. The skin samples were stored at −80 °C, and prior to experimentation they were placed at room temperature for 12 h.

The skin was fixed in a modified Franz vertical percutaneous diffusion cell containing a solution of MTX-mSiO_2_@PDA in 1 mL of PBS (pH 7.4) as the supplying liquid and 20% ethanol solution (pH 5.0, 6.5, or 7.4) as the receptor liquid. At 1, 3, 6, 9, and 12 h, 300 μL samples of the receptor liquid were collected and replaced with the same volume of blank receptor liquid at the same pH. The samples were stored at −20 °C before testing.

### 2.8. In Vivo Pharmacodynamics Study

#### 2.8.1. Establishment of the CIA Model

The DBA/1 mice were randomly divided into two groups, with 6 mice in the normal group and 18 mice in the model group (control group, MTX group and MTX-mSiO_2_@PDA group). The mice were all acclimated and fed for 24 h, and then the ones in the model group were injected with 0.1 mg CΙΙ collagen emulsion in the tail root. The emulsion was prepared by mixing equal volumes of bovine collagen type II (CΙΙ) with complete Freund’s adjuvant (CFA). After 21 days, the mice were administered 0.1 mg of a CΙΙ/IFA (incomplete Freund’s adjuvant) mixture (equal volumes) by subcutaneous injection into the paws. Control group mice were injected with equal volumes of CFA and IFA at days 1 and 21, respectively. All of the mice were observed on a daily basis to monitor the incidence of arthritis.

#### 2.8.2. In Vivo Pharmacodynamic Evaluation

The pharmacodynamics of the established controlled-release MTX delivery system (MTX-mSiO_2_@PDA) were compared to those of the MTX intraperitoneal injections. For this purpose, 10 mg of MTX-mSiO_2_@PDA was dispersed in 5 mL of PBS (pH 7.4). The mixture was sonicated for 5 min, then 50 μL of the suspension was mixed with 200 μL of carbomer 941. Subsequently, 50 μL of the drug-containing gel was applied to one paw of each mouse in the MTX-mSiO_2_@PDA group. Meanwhile, the mice in the MTX group were intraperitoneally injected with 35 μg/g of MTX. The paws of the control group mice were coated with 50 μL of carbomer 941 gel without MTX (pH 7.4).

The thickness of the paws was observed twice a week for four weeks. Then, the mice were sacrificed and their paws were scanned by X-ray to evaluate the pathological indexes of the toe joints. The Skyscan 1174 Micro CT Scanner used to analyze the paws was operated at a voltage, current, and resolution of 50 kV, 800 μA, and 12 μm, respectively, and the field of view was set at 1304 × 1024. The region of interest (ROI) was reconstructed in three dimensions using the heel bone base as a baseline (5.0 mm × 5.0 mm × 6.0 mm in the direction of the toe). The 3D images were reconstructed by the N-Recon software and analyzed by the CT-AN software.

## 3. Results

### 3.1. Synthesis of Nanoparticles

The synthesized materials were characterized by transmission electron microscopy (TEM), dynamic light scattering (DLS), and nitrogen adsorption/desorption analysis (BET).

**Figure 1:**
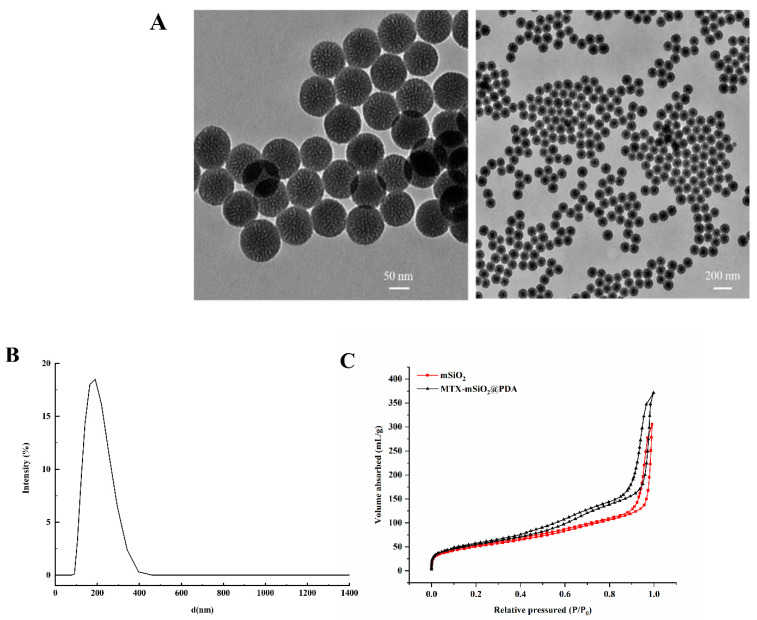

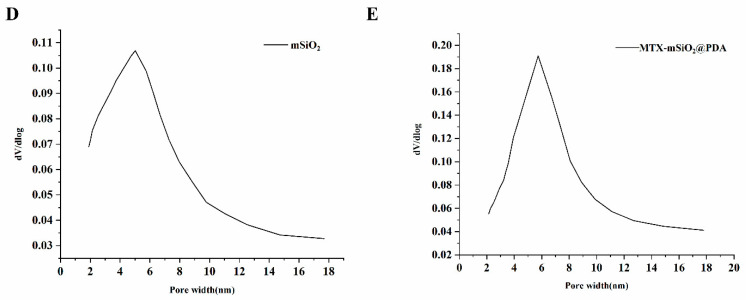
Synthesis of mSiO_2_ and MTX-mSiO_2_@PDA. (**A**) TEM images of mSiO_2_; (**B**) DLS size distribution of mSiO_2_; (**C**) BET of mSiO_2_ and MTX-mSiO_2_@PDA; (**D**) pore size distribution curves of mSiO_2_; (**E**) pore size distribution curves of MTX-mSiO_2_@PDA.

The TEM results shown in Figure 1A demonstrate that the mSiO_2_ particles synthesized herein are uniform in size (about 100 nm), and they were well dispersed, as determined by dynamic light scattering analysis (Figure 1B). The nitrogen adsorption/desorption isotherms presented in Figure 1C indicate that the prepared mSiO_2_ and MTX-mSiO_2_@PDA materials have a mesoporous structure, and, based on the pore size distribution curves (Figure 1D,E), their pore size is about 5 nm. The XPS results (Figure S1) demonstrate the presence of the N element in MTX-mSiO_2_@PDA, a characteristic element of PDA. Overall, these results suggest that biocompatible mesoporous materials may be synthesized by hydrothermal methods.

### 3.2. Analysis of Cellular Uptake

**Figure 2:**
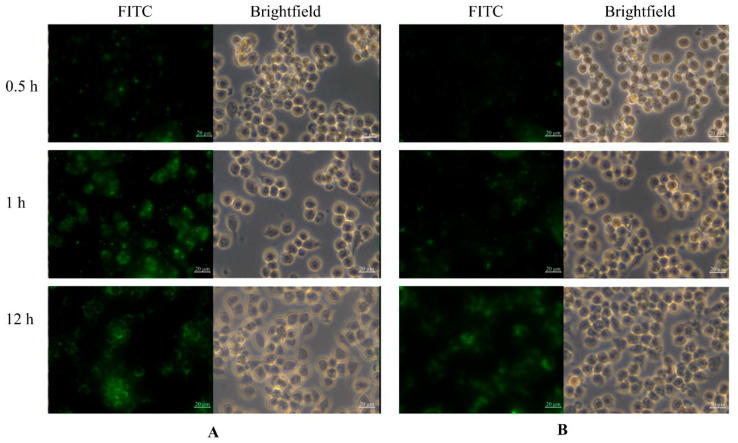
CLSM analysis of cellular (**A**) FITC-mSiO_2_ and (**B**) FITC-mSiO_2_@PDA uptake.

To analyze the cellular uptake of mSiO_2_ and mSiO_2_@PDA, the nanoparticles were labeled with FITC. RAW264.7 cells exposed to the FITC-labeled nanoparticles for 0.5, 1, or 12 h were then observed by confocal laser scanning microscopy (CLSM). As shown in Figure 2, the intracellular fluorescence intensity for both groups (mSiO_2_ and mSiO_2_@PDA) detected at 12 h was remarkably stronger than that observed at 0.5 h. However, the cells administered with MTX-mSiO_2_@PDA exhibited a lower fluorescence intensity than those treated with MTX-mSiO_2_, indicating that the PDA was successfully coated on the surface of mSiO_2_.

### 3.3. In Vitro Antiproliferative Activity

**Figure 3:**
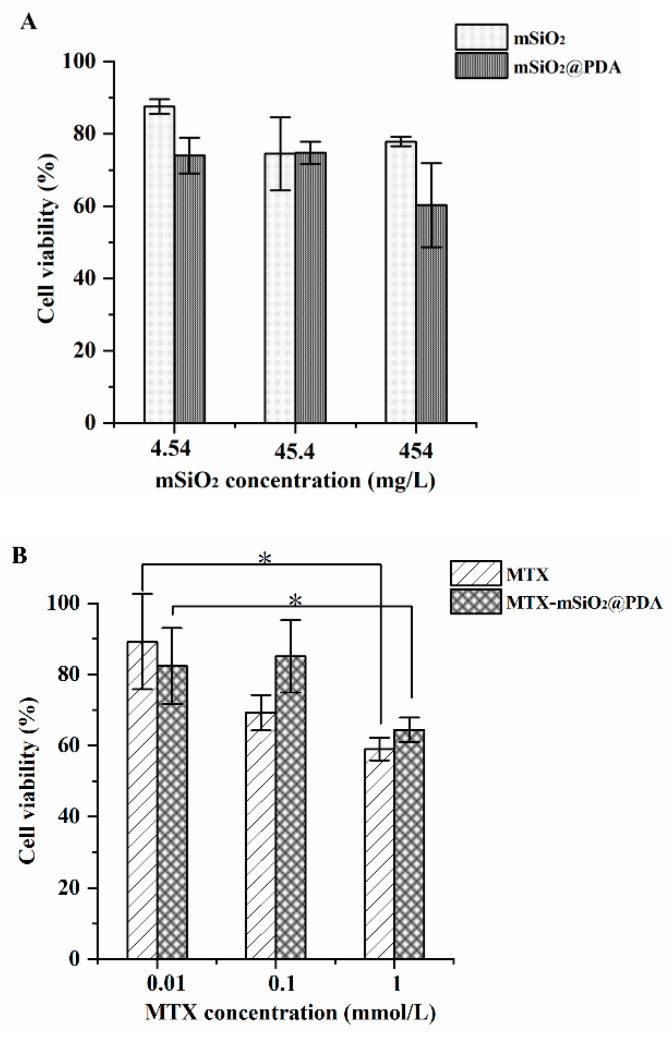
In vitro antiproliferative activities of mSiO_2_, mSiO_2_@PDA, MTX, and MTX-mSiO_2_@PDA. Cytotoxicity of (**A**) mSiO_2_ and mSiO_2_@PDA, (**B**) MTX and MTX-mSiO_2_@PDA in RAW264.7 cells. Data are shown as mean ± SD (n = 3, paired-samples *t*-test * *p* < 0.05).

The cytotoxicity of mSiO_2_, mSiO_2_@PDA, MTX-mSiO_2_, and MTX-mSiO_2_@PDA were explored using RAW264.7 cells. As shown in Figure 3A, mSiO_2_ and mSiO_2_@PDA nanoparticles exhibit no significant cytotoxicity; however, the viability of cells treated with 0.1 mmol/L of MTX decreases by about 30% compared to the cells treated with 0.01 mmol/L of MTX. The 0.1 mmol/L MTX-mSiO_2_@PDA and 0.01 mmol/L MTX-mSiO_2_@PDA cell groups show no obvious variation in cell viability; however, when the concentration of MTX in MTX-mSiO_2_@PDA is increased to 1 mmol/L, the cell viability is decreased to less than 60%, similar to the 1 mmol/L MTX group (Figure 3B). Overall, the results demonstrate that MTX-mSiO_2_@PDA significantly reduces the cytotoxicity of MTX when the concentration of the drug is below 0.1 mmol/L.

### 3.4. pH-Sensitive Drug Release

**Figure 4:**
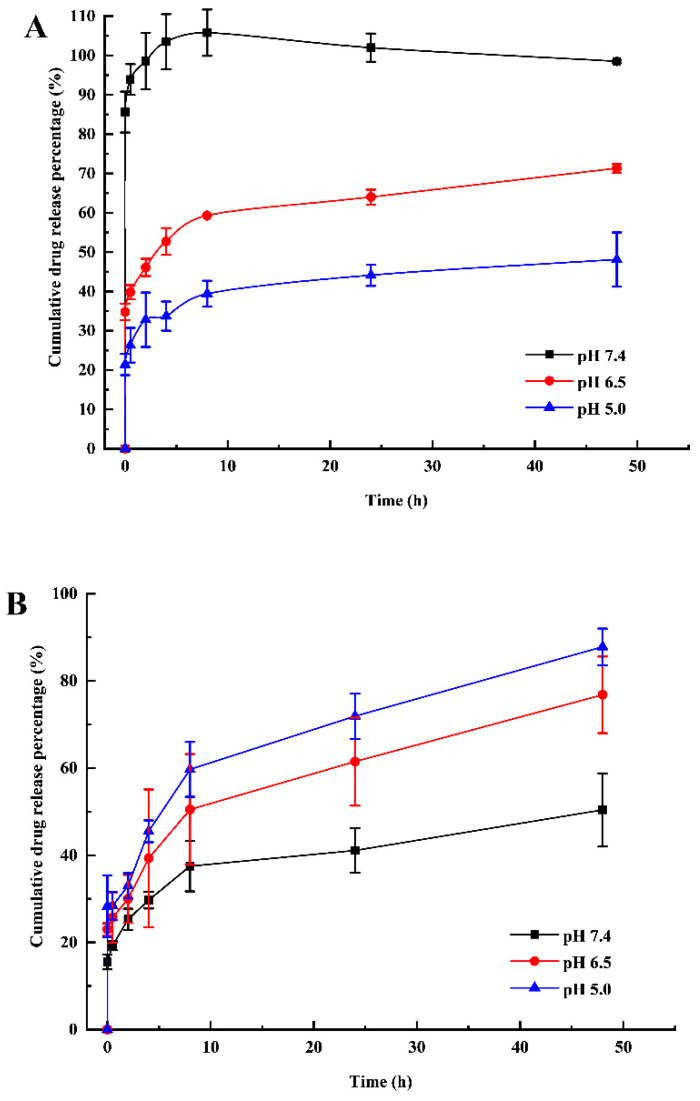
Cumulative drug release under different pH conditions. Percentages of MTX released from (**A**) MTX-mSiO_2_ and (**B**) MTX-mSiO_2_@PDA. Data are shown as mean ± SD (n = 3).

Figure 4A presents the cumulative release percentage of MTX from mSiO_2_ under different pH conditions. At pH 7.4, the amount of drug released after 8 h is about two and three times greater than that released at pH 6.5 and pH 5.0, respectively. Beyond 8 h, no more MTX is released from mSiO_2_, irrespective of the pH.

As shown in Figure 4B, the percentage of cumulative MTX released from mSiO_2_@PDA nanoparticles continuously increases with time (up to 48 h) under all of the investigated pH conditions. Moreover, a greater drug release is detected at lower pH levels. In fact, the cumulative release percentage detected at pH 5.0 is about twice as much as that measured at pH 7.4.

### 3.5. Local Percutaneous Administration

**Figure 5:**
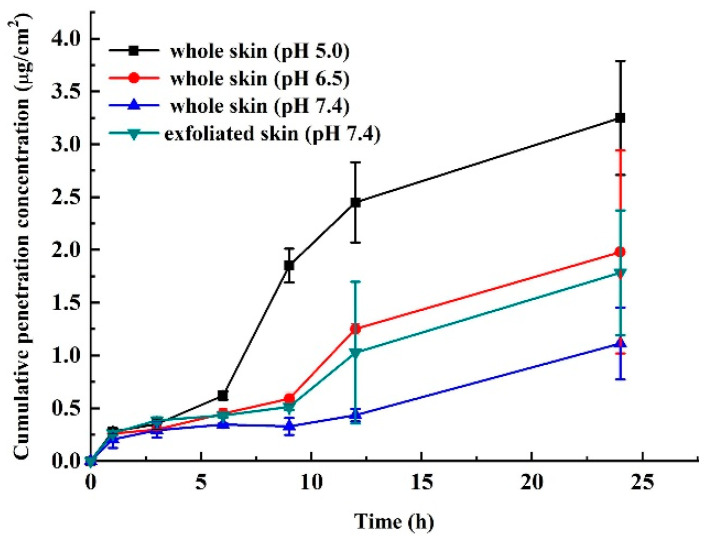
Percutaneous permeation dose-time curves of MTX in MTX-mSiO_2_@PDA measured under different pH conditions. Data are shown as mean ± SD (n = 3).

The amount of MTX released from MTX-mSiO_2_@PDA and that penetrated through the skin after percutaneous administration was investigated under different pH conditions. Interestingly, the cumulative penetration amount increases significantly with decreasing pH in the whole skin, as shown in Figure 5. In fact, the penetrated amount measured at pH 5.0 is three times greater than that measured at pH 7.4. The exfoliated skin exhibits a cumulative penetration amount of MTX that is about twice as great as that detected in the whole skin at the same pH (7.4).

### 3.6. In Vivo Pharmacodynamics

**Figure 6:**
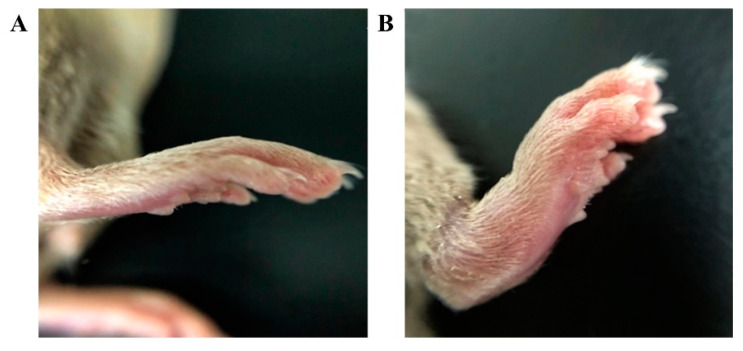
Difference in paw swelling between (**A**) the control group and (**B**) the CIA group.

The CIA model was successfully established in DBA/1 mice. After booster immunization, one or more paws in each mouse showed redness and swelling. The degree of toe swelling in model group mice was significantly greater than that observed in the control group mice, as shown in Figure 6.

**Figure 7:**
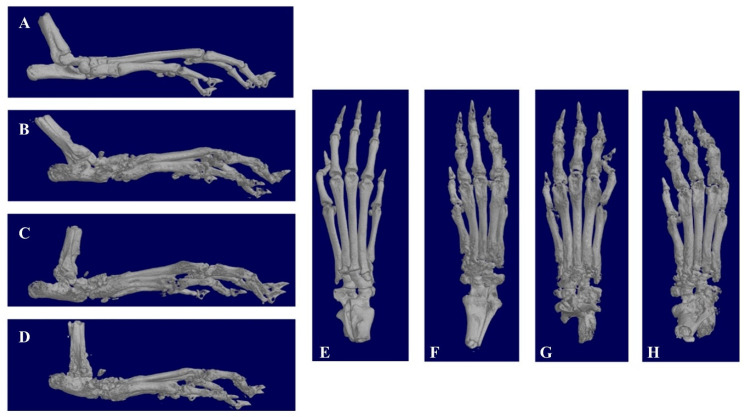
X-ray scans of the paws (**A**,**E**): control group; (**B**,**F**): MTX-mSiO_2_@PDA group; (**C**,**G**): PBS group; (**D**,**H**): MTX intraperitoneal injection group.

The X-ray scans of the paws further confirm that the CIA model had been successfully established. The paws of control group mice are smooth, with a small bone area (127.28 mm^2^). However, the paws of model group mice are rough, and they exhibit severely deformed bone joints. Compared to the control group (paw surface area of 218.87 mm^2^), the surface areas of the paws in mice administered with MTX-mSiO_2_@PDA and MTX intraperitoneal injection are 182.77 and 176.38 mm^2^, respectively (Figure 7).

**Figure 8:**
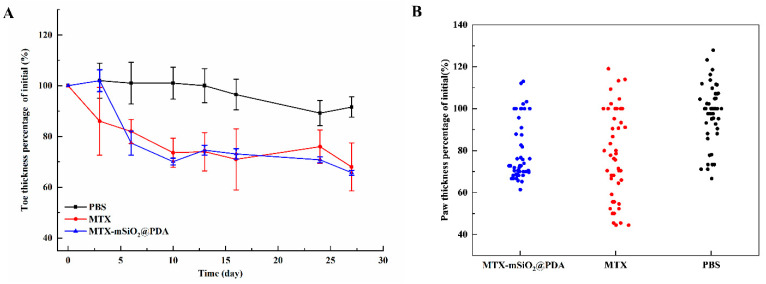
Effect of different modes of MTX administration on the degree of paw swelling (**A**) The variation of toe thickness percentage of initial during 27 days (**B**) The distribution of toe thickness percentage of initial in each group. Data are shown as mean ± SEM (n = 6).

The curative effects of MTX-mSiO_2_@PDA and MTX intraperitoneal injection were also investigated in vivo based on the degree of paw swelling. The obtained results show that both treatments can alleviate paw swelling in mice, and their curative effects are similar. After 27 days, the treatments reduce paw thickness to 70% of the initial value, which is almost equivalent to the normal level (Figure 8A). However, the mice treated with MTX-mSiO_2_@PDA exhibited much smaller toe thickness deviation than those that were administered an MTX intraperitoneal injection (Figure 8B).

## 4. Discussion

In this study, a pH-sensitive, controlled-release drug delivery system was successfully prepared. The system is composed of PDA-modified mSiO_2_ nanoparticles, and it releases the MTX drug under acidic conditions. Considering that the pH in inflammatory joint lesions is less than 7.0, PDA degrades in these lesions, thereby releasing MTX from MTX-mSiO_2_@PDA. The designed drug delivery system increases the curative efficiency of small drug doses and controls the release of the drug, which provides a method for the treatment of rheumatoid arthritis to overcome the side effects of MTX.

Mesoporous silica (mSiO_2_), with a 5.0 nm pore size, was successfully synthesized using hydrothermal synthesis (Figure 1D). At pH 7.4, the drug loading and entrapment rates of this material were 48.4% and 96.8%, respectively (Table S1). Using FITC labeling and confocal laser microscopy, it was shown that MTX-mSiO_2_ nanoparticles can enter cells and deliver MTX (Figure 2). The modification of the MTX-mSiO_2_ nanoparticle surface with PDA significantly decreases the fluorescence intensity of the FITC-labeled mSiO_2_ group, which indicates that the PDA film effectively covers the surface of the nanoparticles. TEM analysis further confirms the successful synthesis of MTX-mSiO_2_@PDA (Figure S4E).

The results of the cell viability assay reveal that mSiO_2_ and mSiO_2_@PDA are biocompatible and not cytotoxic. Moreover, the viability of cells treated with MTX (concentration less than 1.0 mg/L) is more than 60% after 24 h of incubation. Compared with the MTX group, the cytotoxicity of the same concentration of MTX in the MTX-mSiO_2_@PDA group is significantly lower (Figure 3). This indicates that MTX-mSiO_2_@PDA has an excellent biocompatibility.

The designed drug delivery system also exhibits an exceptional controlled release effect. At pH 7.4, MTX is suddenly and effectively released from mSiO_2_ within 8 h (Figure 4A). At lower pH values, the “sudden release” effect is weakened and the cumulative drug release rate of MTX is decreased. This suggests that MTX is only slightly soluble in acidic media, and so it cannot be readily released from mSiO_2_ at pH < 7.0. The nearly stable cumulative release rate achieved after 8 h implies that in the release solution, MTX is under an adsorption/desorption equilibrium. In a physiological environment (pH 7.4), MTX cannot be readily released form PDA-modified mSiO_2_. However, when the pH is decreased, PDA is rapidly degraded, resulting in the effective, controlled release of the drug into the inflammatory tissues. Therefore, PDA modification of mSiO_2_ nanoparticles can be used to avoid the “sudden release” of MTX during local percutaneous administration (Figure 4B).

The local percutaneous administration experiment shows that the main barrier against MTX percutaneous penetration is the stratum corneum, and the cumulative release of MTX increases with the decreasing pH of the receptor liquid (Figure 5).

In addition to the in vitro experiments, the efficiency of the designed drug delivery system was evaluated in vivo using a CIA mouse model (Figure 6, Figure 7 and Figure 8). The obtained results indicate that MTX-mSiO_2_@PDA percutaneous application and MTX intraperitoneal injection exhibit similar therapeutic efficiencies, but compared to the intraperitoneal injection group the experimental deviation of the MTX-mSiO_2_@PDA group is reduced by 20%. These results suggest that the drug delivery system prepared herein enhances the controlled release and therapeutic efficacy of MTX.

## 5. Conclusions

In conclusion, this study is concerned with the design of a pH-sensitive percutaneous system for the delivery of MTX. In this system, polydopamine is used as a “pH-sensitive switch” to control the release of MTX from mSiO_2_ into the joint disease tissue. The results obtained herein indicate that the system effectively increases treatment stability and provide ideas for the future development of percutaneous MTX delivery preparations.

## Data Availability

The original contributions presented in the study are included in the article. Further inquiries can be directed to the corresponding authors.

## Supplementary Materials

The following are available online at https://www.mdpi.com/article/10.3390/nano11112812/s1: Figure S1: XPS spectra of mSiO_2_ and MTX-mSiO_2_@PDA. (A) Wide scan of MTX-mSiO_2_@PDA (B) Wide scan of mSiO_2_ (C) narrow scan of mSiO_2_ N1s peaks and (D) narrow scan of MTX-mSiO_2_@PDA N1s peaks; Figure S2: FT-IR spectra of mSiO_2_, MTX-mSiO_2_, and MTX-mSiO_2_@PDA; Figure S3: MTX standard curve; Figure S4: TEM analysis showing the effect of DA and Tris content ratio on DA polymerization (A) 5 mL Tris + 5 mg DA; (B) 5 mL Tris + 10 mg DA; (C) 10 mL Tris + 5 mg DA; (D) 20 mL Tris + 10 mg DA; (E) TEM images of mSiO_2_-MTX@PDA; Figure S5: Effect of DA polymerization time on MTX release; Table S1: mSiO_2_ loading and entrapment efficiencies at different pH levels.
