# Supplementary material for: Construction of a Nano-Controlled Release Methotrexate Delivery System for the Treatment of Rheumatoid Arthritis by Local Percutaneous Administration
Source: Nanomaterials (Basel). 2021 Oct 23;11(11):2812. doi: 10.3390/nano11112812 (PMC8624172; doi:10.3390/nano11112812)
Supplement: Supplementary file 1 [file nanomaterials-11-02812-s001.zip › nanomaterials-1420929 -SI-done.pdf]

nanomaterials

Supplementary Materials

Construction of a Nano-Controlled Release Methotrexate Delivery System for the Treatment of Rheumatoid Arthritis by Local Percutaneous Administration

Tingting Guo [1,2], Xu Kang [1,2], Sifan Ren [1,2], Xianjin Ouyang [1,2] and Mingming Chang [1,2*]

[1] College of Bioscience and Resource Environment, Beijing University of Agriculture, Beijing 102206, China; g.t0103@icloud.com (T.G.); kangkangkangxu@gmail.com (X.K.); sifanr@outlook.com (S.R.); txiongla0@gmail.com (X.O.)
[2] Key Laboratory of Urban Agriculture (North China) Ministry of Agriculture, Beijing University of Agriculture, Beijing 102206, China
* Correspondence: changmingming@bua.edu.cn; Tel.: +86–010-80765036

1. Synthesis of $mSiO_2$

CTAC (24 mL), water (36 mL), and TEA (0.18 g) were mixed in a flask and magnetically stirred at 60°C for 1 h. A mixture of TEOS (4 mL) with cyclohexane (1 mL) was added to the flask in a dropwise manner, and the solution was stirred slowly at 60°C for 12 h. Subsequently, the obtained particles were washed three times with 0.6% ammonium nitrate ethanol solution in a 60°C water bath, for 6 h each time. Finally, the particles were freeze-dried to obtain $mSiO_2$. The synthesized $mSiO_2$ particles were analyzed by transmission electron microscopy (Figure 1A), dynamic light scattering (Figure 1B), nitrogen adsorption/desorption BET (Figure 1C), XPS (Figure S1), and FT-IR (Figure S2).

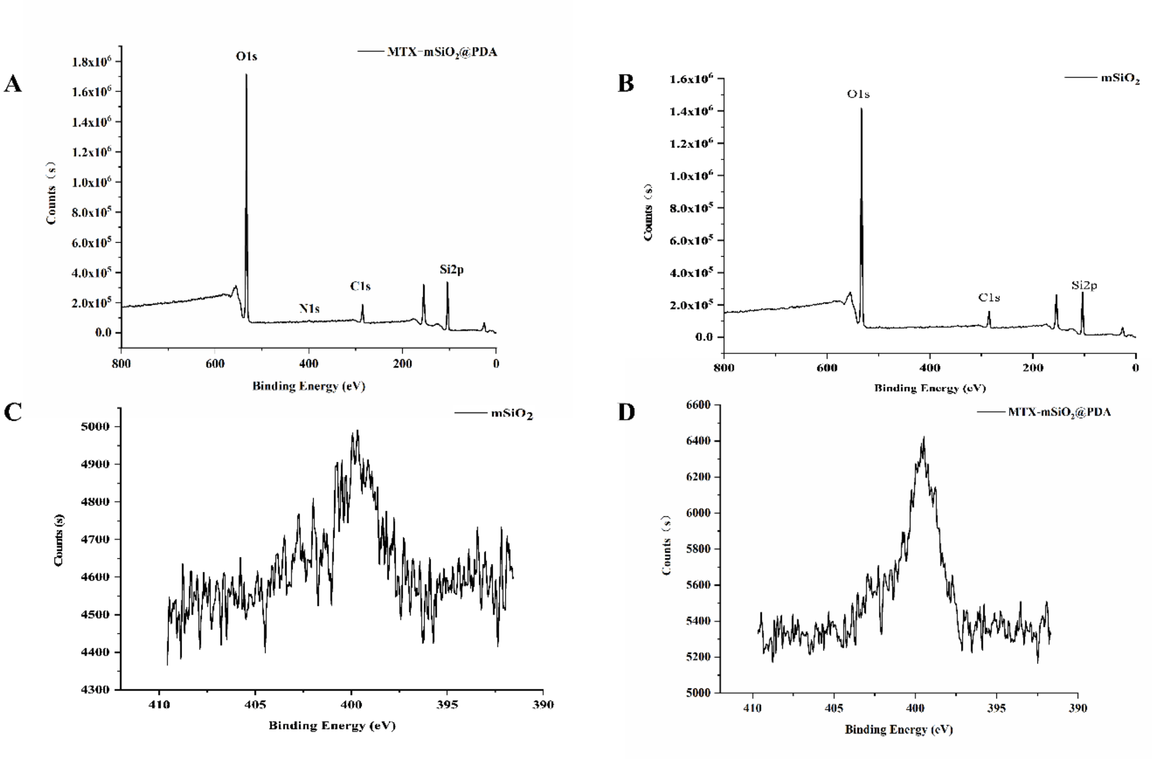

Figure S1. XPS spectra of mSiO2 and MTX-mSiO2@PDA. (**A**) Wide scan of MTX-mSiO2@PDA (**B**) Wide scan of mSiO2 (**C**) narrow scan of mSiO2 N1s peaks and (**D**) narrow scan of MTX-mSiO2@PDA 1s peaks.

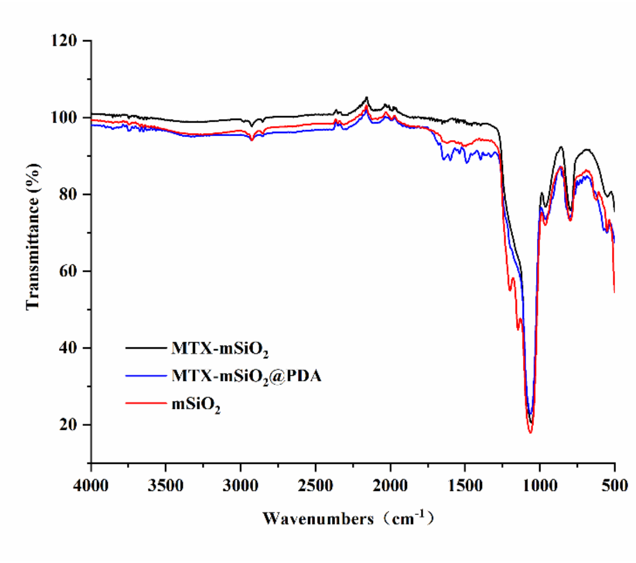

Figure S2. FT-IR spectra of $mSiO_2$, MTX-$mSiO_2$, and MTX-$mSiO_2$@PDA.

2. Loading Experiment

The loading and entrapment efficiencies of MTX in $mSiO_2$ were investigated under different pH conditions, and no significant differences were observed, as shown in Table S1. The loading efficiency ranges between 47.5 and 48.4%, whereas the entrapment efficiency varies between 95.0 and 96.8%.

$$\text{Load efficiency (LE)} = \frac{(MTX_{added} - MTX_{in\ the\ supernatant})}{m_{mSiO_2} + MTX_{added}} \times 100\%$$

$$\text{Entrapment efficiency (EE)} = \frac{(MTX_{added} - MTX_{in\ the\ supernatant})}{MTX_{added}} \times 100\%$$

Table S1. $mSiO_2$ loading and entrapment efficiencies at different pH.

pH	Loading efficiency（%）	Entrapment efficiency（%）
5.0	47.9±0.16	95.7±0.31
6.5	47.5±0.06	95.0±0.13
7.4	48.4±0.03	96.8±0.06

3. Determination of MTX

HPLC analysis (Figure S3) was conducted using a Shimadzu instrument (LC-6AD, Japan) equipped with a SinoPak C18 column (5 μm, 4.6 × 200 mm, Dalian Elite Analytical Instruments Co., Ltd., China). The samples (20 μL) injected into the column were eluted using a mixture of water and acetonitrile (83:17 *v*/*v*) and a solution of 0.1% (*v*/*v*) trifluoroacetic as mobile phases. The detector wavelength was set at 302 nm. A calibration curve was constructed to determine the concentration of MTX in the range of 2.2 to 110 μg/mL (R^2 > 0.999). All of the tests were performed in triplicate.

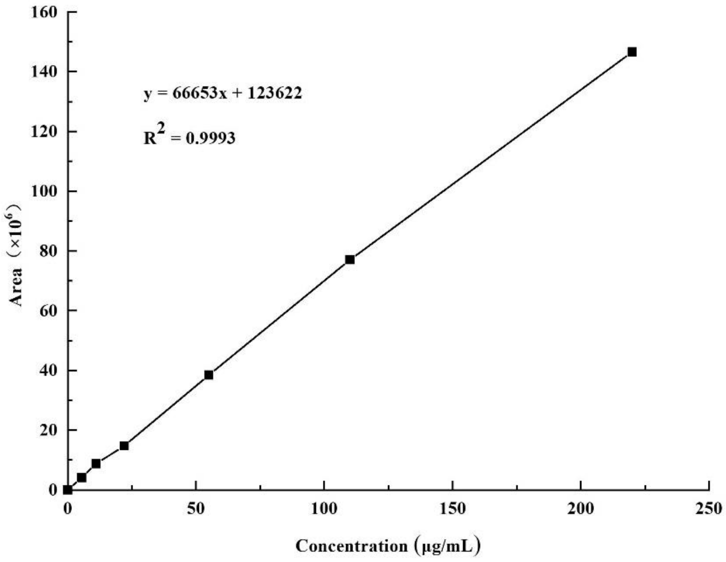

Figure S3. MTX standard curve.

4. Polymerization of DA

DA and MTX-mSiO2 (different ratios) were dispersed in Tris buffer (pH 8.5) and stirred for 1 h. To obtain the MTX-mSiO2@PDA nanoparticles, the unpolymerized DA was washed away by centrifugation in a small amount of Tris buffer at pH 8.5. As shown in Figure. S4, the best coating effect is detected in the group of 5 mL Tris + 5 mg DA. TEM analysis demonstrates that DA can successfully polymerize and uniformly coat the surface of mSiO2.

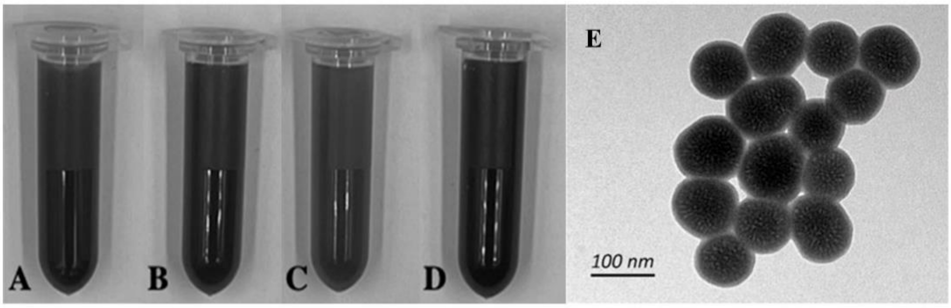

Figure S4. TEM analysis showing the effect of DA and Tris content ratio on DA polymerization (**A**. 5 mL Tris + 5 mg D；**B**. 5 mL Tris + 10 mg DA；**C**. 10 mL Tris + 5 mg DA；**D**. 20 mL Tris + 10 mg DA; **E**. TEM images of mSiO2-MTX@PDA).

To assess the effect of polymerization time on PDA coating efficiency, 5 mg DA and MTX-mSiO2 were dispersed in 5 mL Tris. The results show that the thickness of the PDA coating increases with time. Moreover, thicker PDA coatings significantly improve the "sudden release" of MTX from mSiO2, resulting in a more controlled release of the drug. Based on the results shown in Figure S5, the ideal PDA polymerization time is 24 h.

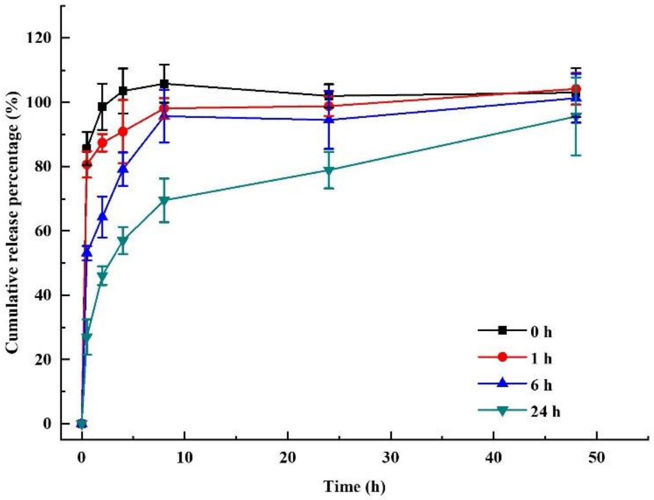

Figure S5. Effect of DA polymerization time on MTX release.
